# Effect of Baduanjin Sequential Therapy on the Quality of Life and Cardiac Function in Patients with AMI After PCI: A Randomized Controlled Trial

**DOI:** 10.1155/2020/8171549

**Published:** 2020-07-04

**Authors:** Ming-Gui Chen, Xuefei Liang, Lili Kong, Jingjing Wang, Fangfang Wang, Xiyan Hu, Jianzhuo He, Rui-Xiang Zeng, Shuai Mao, Liheng Guo, Min-Zhou Zhang, Xiaoxuan Zhang

**Affiliations:** Division of Chest Pain Center, The Second Affiliated Hospital of Guangzhou University of Chinese Medicine, The Second Clinical College of Guangzhou University of Chinese Medicine, Guangzhou 510120, China

## Abstract

**Purpose:**

The purpose of this study was to examine the effects of Baduanjin sequential therapy (BST) on the quality of life and cardiac function in patients with AMI after PCI.

**Subjects:**

96 patients with AMI after PCI were randomly assigned as subjects to two groups: BST group who received 24 weeks of BST training and control group who received no training.

**Methods:**

The methods used in this study included the changes in SF-36 subscales, the measures of left ventricular ejection fraction (LVEF), N-terminal pro-B-type natriuretic peptide (NT-pro-BNP), the body mass index (BMI), and the abdominal circumference.

**Results:**

Of the 96 participants, 82 total patients completed the entire study. At 12 weeks, role physical and health transition of SF-36 were significantly different between the two groups, with a difference of 26.12 (95% CI, 11.59 to 40.64) in role physical and a difference of 15.94 (95% CI, 5.60 to 26.28) in health transition (*p* < 0.05). However, there were statistically significant differences in all aspects of SF-36 between the two groups at 24 weeks (*p* < 0.05). The BST also lowered abdominal circumference and BMI as compared with the control group. In the 24-week follow-up, a significant difference was found in the decline of the LVEF in the control group (*p*=0.020), while there was a nonsignificant difference in the BST group (*p*=0.552). Compared with the control group, the BST group reduced 50 pg/ml on the NT-pro-BNP at 24 weeks (*p*=0.013). The effects of BST exercise were maintained at 24 weeks after the intervention. No serious adverse events were observed.

**Conclusions:**

The BST appears to improve the quality of life in patients with AMI after PCI, with additional benefits of lowered abdominal circumference and BMI and improved level of cardiac function. This trial is registered with NCT02693795.

## 1. Introduction

Acute myocardial infarction (AMI) is one of the leading causes of death around the world, with the potential for substantial morbidity and mortality [1, 2]. From 2001 to 2011, the number of AMI in China increased by four times [3]. Based on the estimation of the Markov model, 21 million new acute coronary events and 7 million cardiac deaths will occur in China in the next 20 years [4]. Although technical advances in percutaneous coronary intervention (PCI) are becoming better and more effective medical therapy, most patients still suffer from angina pectoris, anxiety, depression, and reduced cardiac function after PCI [5–8]. At least 5–10% of survivors die within the first 12 months after the MI and close to 50% need hospitalization within the same year [9, 10].

Previous studies have shown that myocardial ischemia-reperfusion (IR) injury can cause ventricular cell death and is a major pathological event leading to morbidity and mortality in those with acute myocardial infarction [11, 12]. Interestingly, appropriate exercise can rapidly produce a cardiac phenotype that resists IR-induced myocardial injury. It can also relax the heart blood vessels, promote the regeneration of microvessel, and regulate the balance of the autonomic nervous system of the heart [13–15]. As a result, many studies have confirmed that exercise can reduce cardiovascular diseases and significantly decrease the incidence of angina and cardiac events [16, 17]. Exercise also improves patient survival and improves patients' quality of life [18, 19]. However, most exercise programs are complicated and require supervision, and they may even result in an overload of the heart in patients with AMI. A simple easy-to-learn, free-of-charge, and appropriate home-based exercise program is necessary to improve adherence to exercise and help patients with AMI maintain a physically active lifestyle.

Baduanjin exercise, as an important component of Chinese Qigong exercises, is easy to learn and can be performed at home without any assistive equipment [20]. Previous studies have found that Baduanjin exercise is beneficial to health, leading to improvement of strength, physical fitness, and depression [21–23]. However, many studies have focused on the effects of standing Baduanjin on chronic diseases. Baduanjin sequential therapy (BST) which has been developed by our team consists of sitting and standing forms as shown in Supplementary Appendix Figures 1(a) and 1(b). It is simple and effective in clinical practice and the program emphasizes early rehabilitation interventions and continued rehabilitation after discharge. However, there exists few data from large-scale randomized trials that have addressed the effect of Baduanjin exercise on the quality of life and cardiac function in the patient with AMI after PCI. The primary aim of this study was to examine the quality of life and the level of cardiac function in AMI patients after PCI over time in response to a 24-week BST.

## 2. Materials and Methods

### 2.1. Research Design

This study was a parallel, randomized controlled trial (RCT) with a longitudinal research design. Participants were randomly assigned to either the BST group or the control group. Our study strictly complies with the guidelines of the CONSORT 2010 checklist. The study received ethical approval from the Guangdong Provincial Hospital of Chinese Medicine's Human Research Ethics Committee (approval number B2015–41-01), and it was registered with the clinicaltrials.gov (protocol: NCT02693795). This study complies with the principles outlined in the Helsinki Declaration and the relevant regulations of the national regulatory agency.

### 2.2. Participants and Procedures

Recruitment took place in 2016 and 2017, primarily via the screening of hospitalized patients. Eligibility criteria included patients with a clinical diagnosis of AMI, aged 18 to 80 years, performed PCI, and signed an informed consent form. The AMI diagnostic criteria are a reference to the third universal definition of myocardial infarction [24]. Exclusion criteria were current participation in any other behavioral or pharmacological study or instructor-led exercise program, cardiogenic shock, severe heart failure (NYHA cardiac function class IV or LVEF ≤ 35%), malignant arrhythmia (ventricular fibrillation, ventricular tachycardia, and frequent ventricular premature beats), or active bleeding. Patients with poor underlying conditions such as those suffering from bone and joint disease or nervous system disorders that would impede full participation in the study and unavailability during the study period were also excluded.

Patients were randomized to either the BST group or to the control group in a 1 : 1 allocation ratio. Biostatisticians randomized assignments using a sequence randomly generated in the R statistical package. These assignments were put into sealed, opaque envelopes with the date and signature labels placed over the seals of the envelopes. After baseline evaluation of patients, the study coordinator opened the consecutive randomization envelopes and informed the individuals of their group assignments.

Given the nature of the exercise intervention, it was impossible that study participants and exercise instructors could be unaware of the randomization allocation throughout the entire duration of this trial. However, the investigators conducting the outcome assessments and the biostatisticians responsible for the analysis were blinded to the assignment.

The patients in the BST group were asked to complete attendance forms as well as class sign-in sheets for the BST intervention once a month and the patients in the control group would be requested to maintain their original habit of lifestyle. They were also asked to maintain Baduanjin exercise or regular aerobic exercise throughout the follow-up period. Adherence was measured after intervention by telephone calls or WeChat during which research assistants inquired about the frequency and duration of the patients' participation in the BST exercise and general aerobic exercise.

### 2.3. Sample Size

The sample size was calculated on the basis of the changes in SF-36 after 24 weeks of intervention between comparison groups with a significance level of 5% and a two-tailed critical region. According to previous research, the control group can increase the average score of SF-36 by 9 points. Baduanjin can increase the average score of SF-36 by 23 points, and the standard deviation is about 29. According to the formula, *N*=[(*Z*1 − *α*+*Z*1 − *β*)(*s*/Δ)]^2^. The sample size of each group is about 40 cases and a total of 80 cases in the two groups. Considering the situation of dropout and loss of follow-up, it is estimated that the dropout (loss of follow-up) of 20% will require 96 cases.

### 2.4. Interventions

#### 2.4.1. BST Exercise

BST has been developed by our team based on the characteristics of patients with myocardial infarction (Supplementary Appendix Video 1). It consists of the sitting form and the standing form (Supplementary Appendix Figures 1(a) and 1(b)) because the goal is to allow patients with AMI to be also rehabilitated during hospitalization. On the second day after the standard treatment (blood revascularization + drug therapy), patients began the sitting Baduanjin exercises (30 min/session, twice a day, 3 days). After discharge, the patients did the standing Baduanjin exercises (30 min/session, fifth weekly, lasting up to 24-weeks). Patients were provided with a picture-based educational brochure describing the Baduanjin exercises including practicing techniques and safety precautions. Scheduled telephone follow-ups were also provided to reinforce patients' adherence to the 24-week follow-up assessments.

We had strict monitoring indicators and the therapy would be terminated if patients had any of the following conditions. (1) The patient felt laborious and had physical discomforts such as chest pain, dizziness, sweating, and severe dyspnoea. The oxygen saturation was <90%. (2) The FIO_2_ was >60% and breathing was >35 breaths per minute. (3) The systolic blood pressure was <90 or >200 mmHg and mean arterial pressure < 65 mmHg. (4) The patient had unstable heart rhythm or needed to use antiarrhythmic drugs or vasoactive medicines.

### 2.5. Outcome Measures

All outcomes were measured at baseline (week 0) and midterm (week 12) and study completion (week 24). The primary outcome was the SF-36. The secondary outcomes included changes in LVEF, NT-pro-BNP, BMI, and abdominal circumference.

#### 2.5.1. SF-36

The SF-36 health survey scale was developed by the health research institute of New England medical center in Boston, USA, and the Chinese version was successfully developed by the team of Li et al. in 2002, with good reliability and validity [25]. The SF-36 is a widely used, standard instrument for evaluating HRQoL. It is a self-assessment health status questionnaire containing 36 items (questions) about sociodemographic data, health, and personal behavior, grouped into 8 multi-item domains. It measures the following: (1) physical functioning (10 items), (2) social functioning (2 items), (3) role limitations because of physical problems (4 items), (4) role limitations because of emotional problems (3 items), (5) mental health (5 items), (6) energy and vitality (4 items), (7) bodily pain (2 items), and (8) general health perception (5 items). Each of the scores for the domains was coded, summed in an Excel chart, and later transformed in the statistical program SPSS. The results were presented from 0 (worst possible health) to 100 (best imaginable health).

#### 2.5.2. LVEF

The LVEF was obtained through echocardiography. Participants were asked to undergo echocardiographic measurements on the first day of admission, at 12 and 24 weeks after discharge.

#### 2.5.3. NT-pro-BNP

The NT-pro-BNP was determined by sampled blood. All participants were tested for the NT-pro-BNP on admission. The participants were also notified by telephone or WeChat to have blood tests at 12 and 24 weeks after discharge. The laboratory instrument we used was ROCHE, the model was cobae e602, and the reagent batch number was 37537600.

#### 2.5.4. BMI

The BMI is the weight in kilograms divided by the square of the height in meters. Participants were instructed to remove their shoes for measuring their height and weight, and the staff used consistent methods with each patient measurement.

#### 2.5.5. The Abdominal Circumference

The abdominal circumference was measured by tapeline. Patients were asked to stand up and naturally relaxed. The staff measured the abdominal circumference at the navel level 3 times. The results were averaged to derive a range for each measure.

### 2.6. Statistical Analysis

Data were reported as mean and standard deviation for continuous variables and as percentages for categorical variables. All analyses were undertaken in SPSS version 22 (IBM Corp). Independent *t*-tests and chi-square (*χ*^2^) tests were used to examine the homogeneity of demographic and clinical information and the quality of life between the two groups at baseline. Values are presented as mean ± SD and percentage (%) in tables and median with the interquartile range in figures. Repeated measures analysis of variance (ANOVA) was used to test for change over time (i.e., baseline, 12 weeks, and 24 weeks) in the quality of life and NT-pro-BNP for the intervention and control groups. Group comparisons were made by the Mann–Whitney *U* test for continuous variables and the chi-square test for categorical variables. A *p* value of <0.05 was considered significant.

## 3. Results

### 3.1. Characteristics at Baseline between the Two Groups

**Figure 1:**
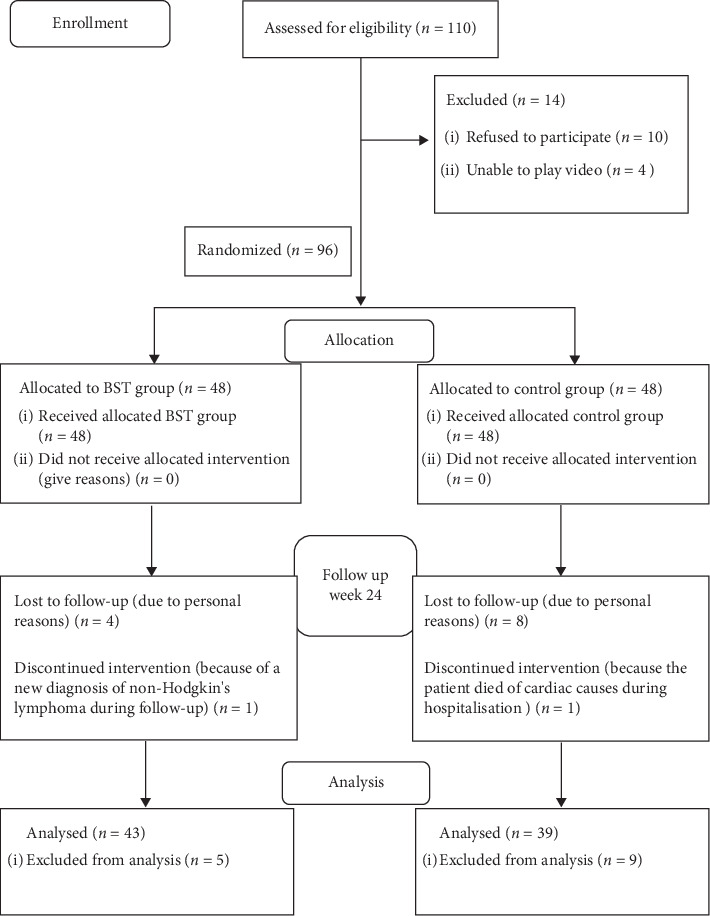
Inclusion and exclusion criteria for this study.

During the study period from July 2016 to August 2017, a total of 110 patients with AMI who underwent PCI were recruited according to the inclusion criteria. Of these patients, 10 refused to participate, and 4 were unable to play videos at home. Most participants who refused to participate in this study did so for personal reasons, including being too busy, having no motivation or interest, and feeling inconvenienced or uncomfortable. 96 patients were randomly assigned to either the BST group (*n* = 48) or the control group (*n* = 48). 14 patients withdrew due to personal reasons (*n* = 12), loss of contact (*n* = 1), and death (*n* = 1). Of the 96 participants, 82 total patients completed the entire study, yielding an attrition rate of 14.58% (Figure 1).

We obtained complete data from 82 cases as shown in Table 1. Table 1 shows the baseline characteristics of the study population. The groups were well matched about baseline characteristics, including age, sex, BMI, NT-pro-BNP, baseline study outcomes of drug treatment, and procedural characteristics (*p* > 0.05).

### 3.2. Primary Outcomes

Mean (±SD) differences between groups in outcome visits at baseline, 12 weeks, and 24 weeks were shown in Table 2. Pairwise comparison and data analysis showed that there was no statistically significant difference between the two groups at the time of enrollment (*p* > 0.05). At 12 weeks, role physical and health transition of SF-36 were significantly different between the two groups, with a difference of 26.12 (95% CI, 11.59 to 40.64) in role physical and a difference of 15.94 (95% CI, 5.60 to 26.28) in health transition (*p* < 0.05). However, there were statistically significant differences in physical functioning, role physical, bodily pain, general health, vitality, social function, role emotional, mental health, and health transition of SF-36 between the two groups at 24 weeks (*p* < 0.05).

### 3.3. Secondary Outcomes

Significant differences between groups were observed after 24 weeks. The BST group had significantly better performance on the abdominal circumference and BMI (Table 3). In the 12 weeks and 24 weeks, the participants in the BST group had a mean decrease of 3.45 centimeters and 4.29 centimeters in abdominal circumference compared with the control group (*p* < 0.05). Regarding BMI, although the 2 groups had no significant improvements during the 12 weeks, there was a considerable improvement in the BST group compared with the control group at 24 weeks (23.40 ± 2.30 and 24.60 ± 2.82, respectively, *p*=0.035).

**Figure 2:**
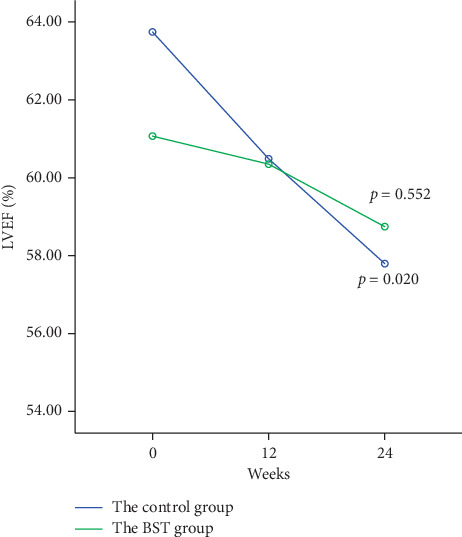
The changes of cardiac function during the 24 weeks. In the 24-week follow-up, the LVEF of the BST group and the control group was baseline (61.06 ± 9.64 and 63.74 ± 8.51, respectively), 12-week outcome (60.34 ± 9.20 and 60.48 ± 9.64, respectively), and 24-week outcome (58.74 ± 10.10 and 57.79 ± 9.56, respectively). A significant difference was found in the decline of the LVEF in the control group (*p*=0.020). A nonsignificant difference was found in the decline of the LVEF in the BST group (*p*=0.552).

**Figure 3:**
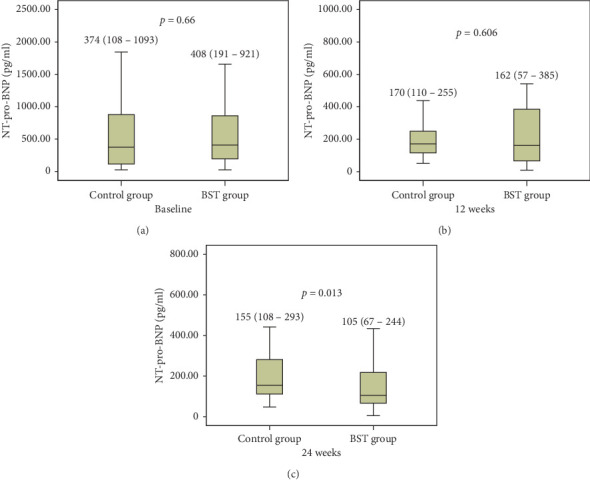
The changes of NT-pro-BNP during the 24 weeks. Values are presented as median with the interquartile range. A significant difference was found in the NT-pro-BNP between the BST and control groups at 24 weeks (*p* < 0.05); a nonsignificant difference was found between the BST and control groups at baseline and 12 weeks (*p* > 0.05).

In the 24-week follow-up, the LVEF of the BST group and the control group was baseline (61.06 ± 9.64 and 63.74 ± 8.51, respectively), 12-week outcome (60.34 ± 9.20 and 60.48 ± 9.64, respectively), and 24-week outcome (58.74 ± 10.10 and 57.79 ± 9.56, respectively) (Figure 2). A significant difference was found in the decline of the LVEF in the control group (*p* < 0.05). A nonsignificant difference was found in the decline of the LVEF in the BST group (*p* > 0.05). Besides, we also found that the BST group and the control group had a significant decrease in the NT-pro-BNP level at the 24-week outcome (105 [67–244], 155 [108–293], respectively, *p*=0.013) (Figure 3);

### 3.4. Adverse Events

During the study period, 1 patient died of cardiac arrest during hospitalization in the control group, and 1 patient withdrew from the study because of a new diagnosis of non-Hodgkin”s lymphoma during follow-up in the BST group. None of these events could be related to the interventions. 4 patients withdrew from the BST group, and 8 patients were removed from the control group for personal reasons.

## 4. Discussion

The results of this study showed that the 24-week BST program was effective for improving the quality of life and cardiac function in patients with AMI after PCI. It could also significantly decrease BMI and abdominal circumference. No serious adverse events were observed during BST training, indicating the safety and usefulness of early intervention for patients with AMI who underwent PCI.

AMI is a common cardiac emergency with a high mortality rate [1]. Although PCI and drugs are currently effective in treating patients, many studies have shown that even after treatment, there is still a high risk of cardiovascular disease recurrence and death after PCI, especially within 1 year after surgery [26, 27]. Therefore, there is considerable concern about this problem. Relevant literature reports that nearly 60% of patients after PCI are severely deficient in exercise, and about 84% of patients after PCI have associated anxiety symptoms, while 67% have associated depressive symptoms [26, 28]. Previous studies have shown that myocardial ischemia-reperfusion (IR) injury can cause ventricular cell death and is a major pathological event leading to morbidity and mortality in those with acute myocardial infarction [11, 12]. Interestingly, appropriate exercise can rapidly produce a cardiac phenotype that resists IR-induced myocardial injury. It is essential for the prognosis of patients with myocardial infarction [13, 15]. Goel and his colleagues counted the proportion of patients with cardiac stents participating in cardiac rehabilitation in a state in the United States and found that poststent exercise rehabilitation had become a trend [8]. Exercise training is beneficial to the patient's cardiac rehabilitation.

To the best of our knowledge, our study is the first RCT to investigate the effect of BST exercise in patients with AMI who underwent PCI. Chen and his colleagues [29] have indicated that Baduanjin exercise can effectively improve the quality of life in patients with heart failure. A meta-analysis of nineteen studies [30] also showed that Baduanjin practice is beneficial for the quality of life. But the benefits of Baduanjin on the quality of life are more evident in older adults and individuals with chronic conditions. In our study, we were studying more severely ill patients with AMI and the results showed that the patients in the BST group performed significantly better than those in the control group at the 24-week outcome visit. Nine aspects of SF-36 were improved compared to the control group. These results were also similar to the findings of Tsang et al. [31].

As we have known, Baduanjin is a low-intensity, aerobic exercise and it is easy to learn because it is less demanding of physical and cognitive skills [32]. Several recent studies have demonstrated other benefits of Baduanjin exercise, including preventing ischaemic stroke, relieving pain and stiffness, and improving sleep quality and psychological well-being [21, 22, 33]. The BST consists of the sitting and the standing Baduanjin. It is developed by our team based on the characteristics of patients with myocardial infarction. The sitting Baduanjin exercise conforms to the aspects of low-intensity and long-term aerobic activity, which is very suitable for the rehabilitation training of patients with AMI after PCI. It was conducted twice a day in the hospital, for 30 min/session. The standing Baduanjin is more suitable for patients with sequential rehabilitation after discharge. Previous studies have shown that the reduction of LVEF following AMI is a result of infarcted myocardium and may involve dysfunctional but viable myocardium [20, 34]. In our study, a nonsignificant difference was found in the decline of the LVEF in the BST group. This shows that BST exercise can prevent the decline of cardiac function. The BST also reduced 50 pg/ml on the NT-pro-BNP at 24 weeks, compared with the control group. Regarding BMI, there was no improvement between the two groups at 12 weeks. However, the participants in the BST group had a mean decrease of 1.21 in BMI compared with the control group at the 24 weeks, which was consistent with a study by An et al. [35], who reported that the BMI values of patients with knee osteoarthritis were significantly improved after one year of Baduanjin exercise. The BST group also had significantly better abdominal circumference after the 12 weeks and 24 weeks of intervention. The results of the analysis indicated that prolonged BST exercise was of benefit to the health of the patients with AMI who underwent PCI.

The performance of BST can systematically enhance cardiopulmonary functions and concurrently modulate mind and spirit, ultimately achieving the integration of the mind and body. However, the mechanisms behind the therapeutic change remain less understood and warrant future exploration.

### 4.1. Study Limitations

This study has some limitations. First, this is a single-center study. Second, because they were given behavior-based treatments, the participants were aware of their intervention assignments. Moreover, we are unable to provide a definitive physiological mechanism for the effects of the Baduanjin exercise. Nonetheless, this study offers informative data from the first large-scale clinical trial of BST in patients with AMI after PCI.

## 5. Conclusions

The BST appears to improve the quality of life in patients with AMI after PCI, with additional benefits of lowered abdominal circumference and BMI and improved level of cardiac function.

## Figures and Tables

**Table 1:** Baseline clinical and procedural characteristics.

Variable	Control group (*n* = 39)	BST group (*n* = 43)	*p* value
*Clinical characteristics*			
Age (years)	61.49 ± 11.54	59.98 ± 10.86	0.543
Male, *n* (%)	30 (76.9)	29 (67.4)	0.34
Body mass index (kg/m^2^)	24.62 ± 2.86	23.95 ± 2.35	0.25
Hypertension, *n* (%)	22 (56.4)	26 (60.5)	0.71
Dyslipidaemia, *n* (%)	11 (28.2)	14 (32.6)	0.669
History of smoker, *n* (%)	25 (64.1)	22 (51.2)	0.237
History of CAD, *n* (%)	1 (2.6)	6 (14)	0.148
Diabetes, *n* (%)	10 (25.6)	9 (20.9)	0.614
LVEF (%)	63.74 ± 8.52	61.07 ± 9.64	0.189
LVEDD (mm)	46.54 ± 3.73	44.83 ± 4.68	0.073
LVESD (mm)	29.77 ± 5.52	29.82 ± 5.71	0.97
NT-pro-BNP (pg/ml)	374 (108–1093)	408 (191–921)	0.66
WBC (10^9^/L)	10.55 ± 3.12	10.97 ± 3.51	0.574
TG (mmol/L)	1.51 (1.09–2.19)	1.53 (1.10–2.30)	0.795
TC (mmol/L)	4.86 ± 0.85	4.87 ± 1.13	0.971
HDL-C (mmol/L)	1.10 ± 0.28	1.12 ± 0.44	0.856
LDL-C (mmol/L)	3.38 ± 0.78	3.53 ± 0.92	0.425

*Drug treatment*			
Statins, *n* (%)	39 (100)	43 (100)	
Aspirin, *n* (%)	39 (100)	43 (100)	
Clopidogrel, *n* (%)	39 (100)	43 (100)	
b-Blockers, *n* (%)	16 (41)	20 (46.5)	0.617
Calcium blockers, *n* (%)	6 (15.4)	5 (11.6)	0.618
ACE inhibitors, *n* (%)	6 (15.4)	3 (7)	0.388
ARBs, *n* (%)	6 (15.4)	14 (32.6)	0.71
Diuretics, *n* (%)	4 (10.3)	3 (7)	0.893

*Procedural characteristics*			
Transradial access, *n* (%)	36 (92.3)	39 (90.7)	1

*Target vessel*			
Left main	1 (2.6)	1 (2.6)	1
Left anterior descending	32 (82.1)	33 (76.7)	0.554
Left circumflex	20 (51.3)	19 (44.2)	0.521
Right coronary artery	23 (59)	25 (58.1)	0.939

*Lesion location*			
Proximal	24 (61.5)	29 (67.4)	0.577
Middle	27 (69.2)	27 (62.8)	0.539
Distal	4 (10.3)	7 (16.3)	0.424
Branch	13 (33.3)	13 (30.2)	0.763
Preoperative TIMI 0	17 (43.6)	18 (41.9)	0.874
Preoperative TIMI 1	2 (5.1)	1 (2.3)	0.931
Preoperative TIMI 2	1 (2.6)	2 (4.7)	1
Preoperative TIMI 3	19 (48.7)	22 (51.2)	0.825
Postoperative TIMI 3	39 (100)	43 (100)	

*Lesion classification*			
Number of stents implanted	1.18 ± 0.389	1.09 ± 0.294	0.263

BMI, body mass index; CAD, coronary artery disease; NT-pro-BNP, N-terminal pro-brain natriuretic peptide; TG, triglyceride; TC, total cholesterol; HDL-C, high-density lipoprotein cholesterol; LDL-C, low-density lipoprotein cholesterol; ACE, angiotensin-converting enzyme; ARBs, angiotensin receptor blockers. The BMI is the weight in kilograms divided by the square of the height in meters. Values are expressed as the mean ± SD, the median with interquartile range, or *n* (%). *p* values were calculated by *t*-test for continuous variables and by the chi-square test or Fisher's exact test for categorical variables.

**Table 2:** The changes of SF-36 subscales during the 24 weeks.

Measure	Control group (*n* = 39)	BST (*n* = 43)	BST vs. control group (95% CI)	*p* value
*Physical functioning*				
Baseline	77.05 ± 17.15	71.51 ± 17.27	−5.54 (−13.12 to 2.04)	0.150
12 weeks	80.13 ± 16.60	86.33 ± 10.73	6.20 (0.11 to 12.28)	0.051
24 weeks	82.69 ± 12.34	90.11 ± 10.72	7.42 (2.35 to 12.49)	0.005

*Role physical*				
Baseline	35.89 ± 32.34	34.88 ± 38.76	−1.01 (−16.79 to 14.76)	0.899
12 weeks	44.23 ± 33.67	70.34 ± 32.39	26.12 (11.59 to 40.64)	0.001
24 weeks	69.87 ± 40.63	90.11 ± 16.49	20.24 (6.85 to 33.64)	0.004

*Bodily pain*				
Baseline	61.90 ± 21.61	56.44 ± 25.09	−5.46 (−15.80 to 4.88)	0.297
12 weeks	80.97 ± 17.70	79.30 ± 13.59	−1.67 (−8.57 to 5.23)	0.631
24 weeks	80.35 ± 19.89	90.05 ± 11.81	9.69 (2.58 to 16.80)	0.008

*General health*				
Baseline	56.92 ± 15.08	52.00 ± 14.33	−4.92 (−11.38 to 1.54)	0.134
12 weeks	65.28 ± 16.27	59.58 ± 19.81	−5.70 (−13.72 to 2.31)	0.161
24 weeks	63.54 ± 18.73	77.84 ± 16.19	14.30 (6.62 to 21.98)	<0.001

*Vitality*				
Baseline	59.23 ± 14.31	55.58 ± 12.21	−3.65 (−9.48 to 2.18)	0.216
12 weeks	62.18 ± 14.99	67.21 ± 9.96	5.03 (−0.64 to 10.70)	0.081
24 weeks	62.31 ± 8.57	73.95 ± 8.42	11.65 (7.91 to 15.38)	<0.001

*Social function*				
Baseline	83.97 ± 24.15	77.61 ± 26.10	−6.36 (−17.45 to 4.73)	0.257
12 weeks	71.28 ± 24.62	72.56 ± 20.94	1.28 (−8.74 to 11.29)	0.801
24 weeks	71.79 ± 24.37	82.32 ± 12.87	10.53 (1.78 to 19.28)	0.019

*Role emotional*				
Baseline	61.53 ± 42.26	47.98 ± 38.04	−13.55 (−31.20 to 4.09)	0.130
12 weeks	69.23 ± 40.74	81.39 ± 25.51	12.16 (−3.02 to 27.34)	0.114
24 weeks	70.08 ± 38.08	96.12 ± 10.81	26.04 (13.31 to 38.77)	<0.001

*Mental health*				
Baseline	71.28 ± 16.51	66.32 ± 17.07	−4.96 (−12.35 to 2.44)	0.186
12 weeks	78.97 ± 15.78	80.19 ± 13.38	1.21 (−5.20 to 7.62)	0.708
24 weeks	74.87 ± 15.27	82.23 ± 17.38	7.36 (0.14 to 14.58)	0.046

*Health transition*				
Baseline	36.54 ± 26.19	44.19 ± 24.90	7.65 (−3.58 to 18.87)	0.179
12 weeks	44.87 ± 22.34	60.81 ± 24.49	15.94 (5.60 to 26.28)	0.003
24 weeks	51.28 ± 22.18	82.56 ± 19.31	31.28 (21.99 to 40.56)	<0.001

Data are presented as mean ± SD. *p* values were calculated by *t*-test for continuous variables.

**Table 3:** The changes of abdominal circumference and BMI during the 24 weeks.

Measure	Control group (*n* = 39)	BST (*n* = 43)	BST vs. control group (95% CI)	*p* value
*Abdominal circumference*				
Baseline	90.84 ± 9.44	87.87 ± 6.68	−2.96 (−6.61 to 0.68)	0.109
12 weeks	90.34 ± 7.00	86.89 ± 5.25	−3.45 (−6.25 to −0.65)	0.016
24 weeks	90.26 ± 7.10	85.98 ± 5.79	−4.29 (−7.18 to −1.39)	0.004

*BMI*				
Baseline	24.62 ± 2.86	23.95 ± 2.35	−0.67 (−1.81 to 0.48)	0.249
12 weeks	24.57 ± 2.77	23.94 ± 2.33	−0.63 (−1.75 to 0.49)	0.268
24 weeks	24.60 ± 2.82	23.40 ± 2.30	−1.21 (−2.34 to −0.09)	0.035

BMI, body mass index. Data are presented as mean ± SD.

## Data Availability

The data used to support the findings of this study are available from the corresponding author upon request.

## Supplementary Materials

Supplementary Materials Supplementary Appendix Figure 1: (a) the introduction of the sitting Baduanjin exercises by the intensive care unit of Guangdong Provincial Hospital of Chinese Medicine. (b) the introduction of the standing Baduanjin exercises by the intensive care unit of Guangdong Provincial Hospital of Chinese Medicine. Supplementary Appendix Video 1: cardiac rehabilitation exercises of the Baduanjin sequential therapy.
