# Supplementary material for: Effect of Baduanjin Sequential Therapy on the Quality of Life and Cardiac Function in Patients with AMI After PCI: A Randomized Controlled Trial
Source: Evid Based Complement Alternat Med. 2020 Jul 4;2020:8171549. doi: 10.1155/2020/8171549 (PMC7355341; doi:10.1155/2020/8171549)

Supplementary Appendix figure 1

（a）The introduction of the sitting Baduanjin exercises by the intensive care unit of Guangdong provincial hospital of Chinese medicine

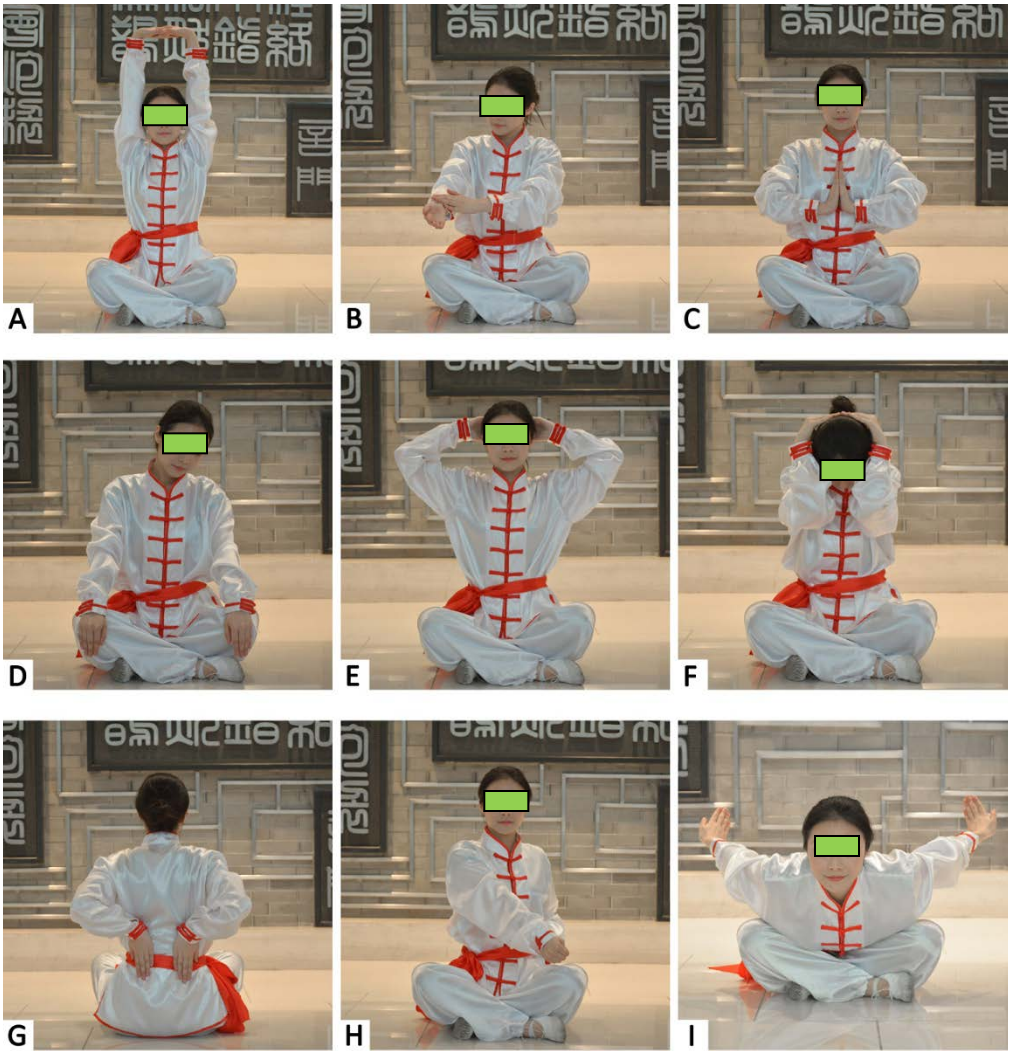

(b) The introduction of the Standing Baduanjin exercises by the intensive care unit of Guangdong provincial hospital of Chinese medicine.

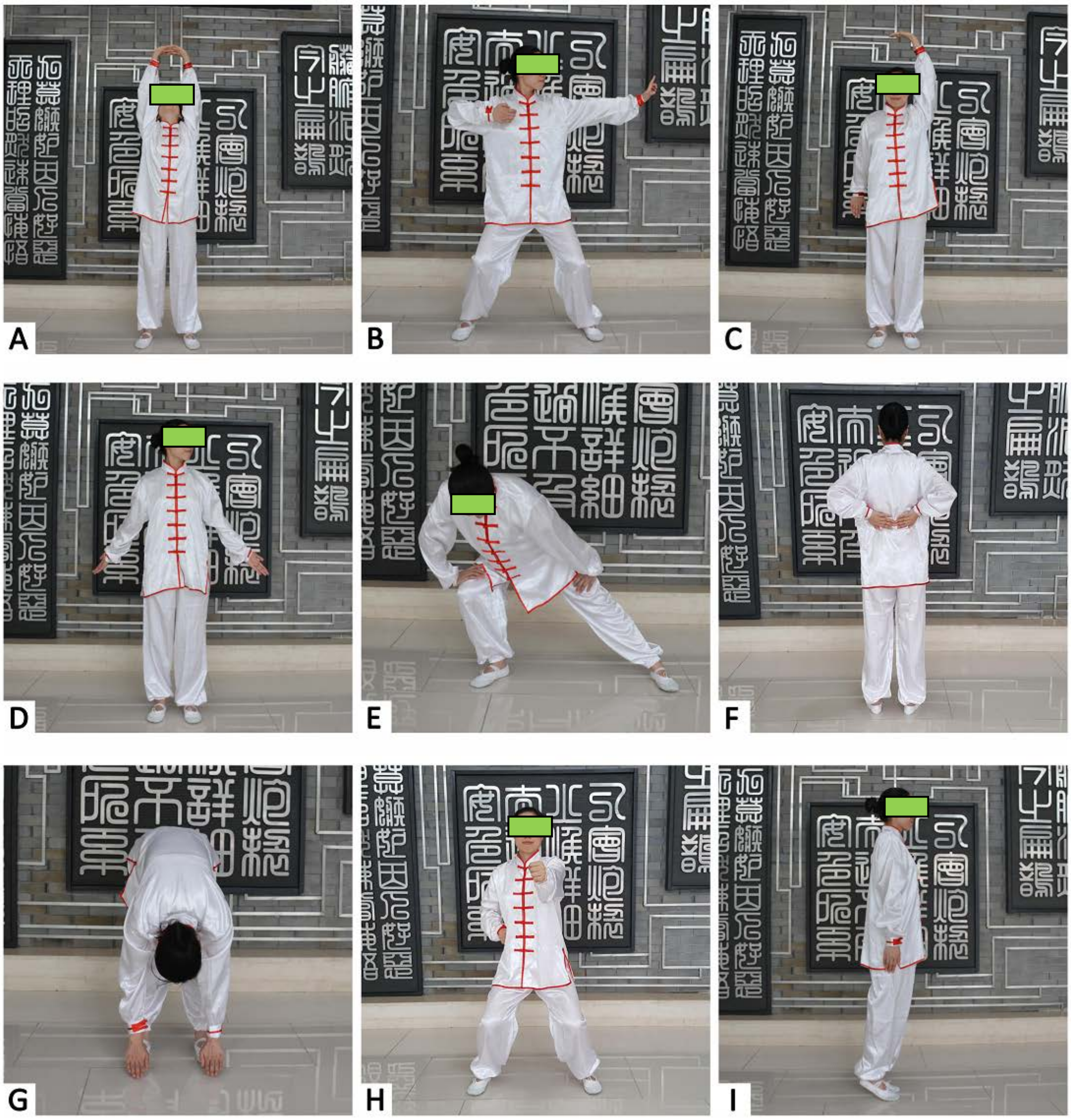

Supplement: Supplementary Materials — Supplementary Appendix Figure 1: (a) the introduction of the sitting Baduanjin exercises by the intensive care unit of Guangdong Provincial Hospital of Chinese Medicine. (b) the introduction of the standing Baduanjin exercises by the intensive care unit of Guangdong Provincial Hospital of Chinese Medicine. Supplementary Appendix Video 1: cardiac rehabilitation exercises of the Baduanjin sequential therapy. [file 8171549.f1.zip › 8171549.f1/Supplementary Appendix figure1.pdf]
